# Genomic Structure and Evolution of Multigene Families: “Flowers” on the Human Genome

**DOI:** 10.1155/2012/917678

**Published:** 2012-06-19

**Authors:** Hie Lim Kim, Mineyo Iwase, Takeshi Igawa, Tasuku Nishioka, Satoko Kaneko, Yukako Katsura, Naoyuki Takahata, Yoko Satta

**Affiliations:** ^1^Center for the Promotion of Integrated Sciences, The Graduate University for Advanced Studies (SOKENDAI), Hayama, Kanagawa 240-0193, Japan; ^2^Department of Biochemistry and Molecular Biology, Pennsylvania State University, 312 Wartik Laboratory, University Park, PA 16802, USA; ^3^Institute for Amphibian Biology, Graduate School of Science, Hiroshima University, Higashihiroshima, Hiroshima 739-8526, Japan; ^4^Laboratory of Plant Genetics, Graduate School of Agriculture, Kyoto University, Kyoto 606-8502, Japan; ^5^Department of Evolutionary Studies of Biosystems, The Graduate University for Advanced Studies (SOKENDAI), Hayama, Kanagawa 240-0193, Japan; ^6^The Graduate University for Advanced Studies (SOKENDAI), Hayama, Kanagawa 240-0193, Japan

## Abstract

We report the results of an extensive investigation of genomic structures in the human genome, with a particular focus on relatively large repeats (>50 kb) in adjacent chromosomal regions. We named such structures “Flowers” because the pattern observed on dot plots resembles a flower. We detected a total of 291 Flowers in the human genome. They were predominantly located in euchromatic regions. Flowers are gene-rich compared to the average gene density of the genome. Genes involved in systems receiving environmental information, such as immunity and detoxification, were overrepresented in Flowers. Within a Flower, the mean number of duplication units was approximately four. The maximum and minimum identities between homologs in a Flower showed different distributions; the maximum identity was often concentrated to 100% identity, while the minimum identity was evenly distributed in the range of 78% to 100%. Using a gene conversion detection test, we found frequent and/or recent gene conversion events within the tested Flowers. Interestingly, many of those converted regions contained protein-coding genes. Computer simulation studies suggest that one role of such frequent gene conversions is the elongation of the life span of gene families in a Flower by the resurrection of pseudogenes.

## 1. Introduction

A genomic structure is a region of repeats located on adjacent chromosomal regions and consists of combinations of tandem or/and inverted repeats and palindromes. Genomic structures are generated by genomic rearrangements such as duplications, deletions, and inversions in a genome. If the genes are located within a rearrangement such as a duplication or deletion, the number of genes would vary between individuals, and consequently, expression levels of those genes could also vary [1, 2]; this may thus affect phenotypic traits. Well-known inherited diseases, such as Prader-Willi and Williams-Beurens syndromes, are caused by variation in the gene numbers and, in particular, by deletions in 15q11–q13 and 7q11.23, respectively [3]. On the other hand, inversions do not result in changes in the copy number of genes, but could affect recombination frequency between an intact and inverted segment (haplotype). Recombination is suppressed, and therefore, both haplotypes accumulate specific mutations. This accumulation enhances genetic differentiation between genomes within a species. If genes in the recombination-suppressed region are involved in mating or adaptation to environmental changes, the inversion might affect reproductive isolation or speciation [4].

Genomic structures can be detected by comparing nucleotide sequences within an individual genome. Such structures are not distributed uniformly in a genome and sometimes are located on particular regions in a chromosome. One well-known example is a cluster of palindromes on the Y chromosome [5]. There are eight palindromes of various sizes, and six of them carry protein-coding genes. The palindromes have been maintained via genetic exchanges between arms within a palindrome or between different palindromes [6]. These palindromes are important in keeping a large number of copies of male-specific Y chromosomal genes identical [7].

Duplication occurring in the genome is often called “segmental duplication,” which is distinct from whole genome duplication. There have been several studies involving screening of segmental duplications collectively for the entire human genome [8, 9]. Within human populations, copy number variations (CNVs) in segmental duplications or genomic structures have been found [8, 9]. Massive structural variations were reported [8, 9], but the biological significance of the frequent genomic rearrangements during evolution remains unclear. It is thus necessary to shed light on each region to understand the evolution of genomic rearrangements.

The aim of this study was to reveal how rearrangements in the genome or genomic structures have been maintained and to determine the biological significance of their retention, from an evolutionary point of view. Large-scale duplicated sequences are amongst the most difficult sequences to assemble. For our study, the quality of assembly is most important. In this regard, the human genome sequence is the most reliable. Thus, we have studied the evolution of the genomic structures on the human genome.

## 2. Materials and Methods

### 2.1. Identification and Statistical Analyses of Flowers

Human genome sequence data (build 36; 2,858,142,293 bp) and corresponding gene information was obtained from NCBI (http://www.ncbi.nlm.nih.gov/). To detect genomic structures (such as an inversions, tandem repeats, or deletions/insertions), dot-matrix and BLAST analyses using the software package *GenomeMatcher* [10] were applied to the genome sequence. *GenomeMatcher* was used to search for homologous sequences between two input sequences and to visualize the results as dot plots. To detect regions where at least two duplications are located in close proximity, we first divided genomic sequences into 1-Mb nonoverlapping regions. In each of these regions, we compared sequences by itself using BLAST with a command line “*blastn-F T-W 40-e 0.01-D 1*.”

The results of “BLAST hits” for each region are alignments of pairs of the query and subject sequences. Our method focused on identifying genomic structures, which are duplication-concentrated regions generated by genomic rearrangements. A “Flower” is composed of an aligned sequence of more than 1 kb, excluding the concentrated regions of repetitive elements, such as LINEs, SINEs, and microsatellites. The size of a Flower, that is, the length of the region containing the alignments, was selected as longer than 50 kb. In the case of duplications located across neighboring windows, we ran BLAST for extended windows in order to detect large-sized Flowers. If the location of two duplications was not within 1 Mb in length, our detection method could miss such regions, but they were not of interest to us.

**Figure 1:**
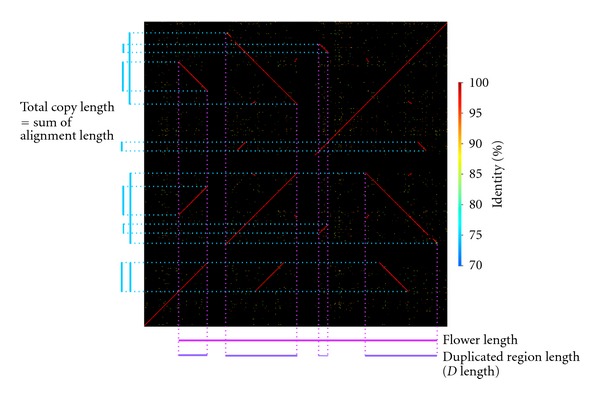
Example of a Flower. A dot plot shows an example of a Flower located on chromosome 10. Diagonal lines in the plot indicate the positions of blast hits, and the colors of the lines represent the identity of alignments as shown in the right-hand side of the plot. A blue bar indicates a detected copy unit, and the sum of alignments of blast hits equals the sum of the copy length. A pink bar indicates a Flower region, and the length of the bar represents the length of the Flower. A purple bar indicates a region called the duplicated region that contains the copy.

 One example of a Flower is shown in Figure 1. Several measurements for each Flower were defined (Figure 1, Table 1). The start and end points of a Flower were determined by BLAST analysis. In the example in Figure 1, *Flower length* was defined as the consecutive segment size from the start to the end point of a Flower. The *D length* for a Flower was defined as the sum of lengths of duplicated regions in a Flower. The effective number of copy units was calculated by the ratio of the total length of aligned sequences to the *D* length. *Copy unit length* was defined by the *D* length/the effective number of copy units. *Length of inverted copy* was defined as the total length of sequences matched to the query sequences in an opposite direction. The *minimum and maximum identity* were defined as the minimum and maximum values among identities of all alignments in a single Flower, respectively. The *gene region length over D length* stands for the proportion of gene regions in a repeated region, defined as the *gene number* in a Flower. When the number is more than 0.1, the gene was determined to be a “Flower gene”.

### 2.2. Association of Flowers with Genes

To examine the relationship between Flowers and the genes in them, we compared gene density (measured by the number of genes per 1 Mb) in the duplicated regions of Flowers with the density of genes in randomly selected regions of the genome. Among 291 Flowers, there were 277 Flowers whose *D* lengths were unique. We sampled 277 regions of same sizes from the human genome, with the exception of Flower regions and sequencing gaps. Based on the annotation data of NCBI build 36, the number of genes in non-Flower and nongap regions was counted. Thousands of sampling repeats were performed to obtain the distribution of the gene number in such regions.

 To investigate the known or reported functions of Flower genes, we extracted the Gene Ontology (GO) ID and obtained information on the GO categories from data of human genes at NCBI (http://www.ncbi.nlm.nih.gov/) and the GO database (http://www.geneontology.org/; The Gene Ontology Consortium).

### 2.3. Gene Conversion Detection Test

To detect gene conversion events within Flowers, we used the CHAP package [12]. To run the package, at least one orthologous sequence for each Flower must be determined as an outgroup. We performed BLAST with queries of Flower sequences and subjects of chimpanzee or gorilla genome assemblies. The identification of orthologs was difficult for large-sized Flowers or Flowers on sex chromosomes because of the quality of those assemblies. For accurate detection of gene conversion, we analyzed only Flowers for which orthologs were clearly determined. Finally, we succeeded in obtaining 189 Flowers with their orthologous sequences. We counted only conversion events occurring between human paralogous sequences after split with chimpanzees or gorilla.

### 2.4. Simulation Analyses

Our simulation model assumed two loci in a diploid population with an effective size of *N* = 50. Although *N* = 50 could be considered as being too small given the size of the human population (*N* = 10^4^), under neutrality, the simulation can be scaled while keeping the population parameters *Nμ* and *Nc* constant.

We set generation 1 as the time when a duplicated identical gene is fixed in a population. Mutations occurred at a rate of *Nμ* = 0.5 per locus per generation, and each mutation caused pseudogenization. A backward mutation was not allowed and infinite sites model for mutations was used. Gene conversion occurred from one gene to another at a rate of *Nc* = 0–20 per gene per generation. Since we assume two loci with four genes in a diploid individual, the conversion can occur in any of three different schemes: allelic trans, allelic cis, and nonallelic trans (Figure 4). The number of pseudogenes (*n*_*ψ*_) in a diploid individual ranges from 0 to 4. When *n*_*ψ*_ was 0 to 3, no natural selection against an individual having the pseudogene(s) was considered (*Ns* = 0, where *s* is a selection coefficient), whereas when *n*_*ψ*_ in an individual becomes 4, there is negative selection against the individual with *Ns* = 2–5. In one generation, simulations were performed according to the following order: mutation in a gamete → gene conversion between gametes in an individual → random sampling of gametes → selection against a progeny (zygote). A simulation counted the number of generations from generation 1 until all genes at both loci in a population became pseudogenes (fixation of pseudogenes). We carried out 10,000 repeats of this simulation and calculated the mean and standard error of this fixation time of 10,000 replications for each set of parameters (i.e., *Nc* and *Ns*).

## 3. Results

### 3.1. 291 Flowers

**Figure 2:**
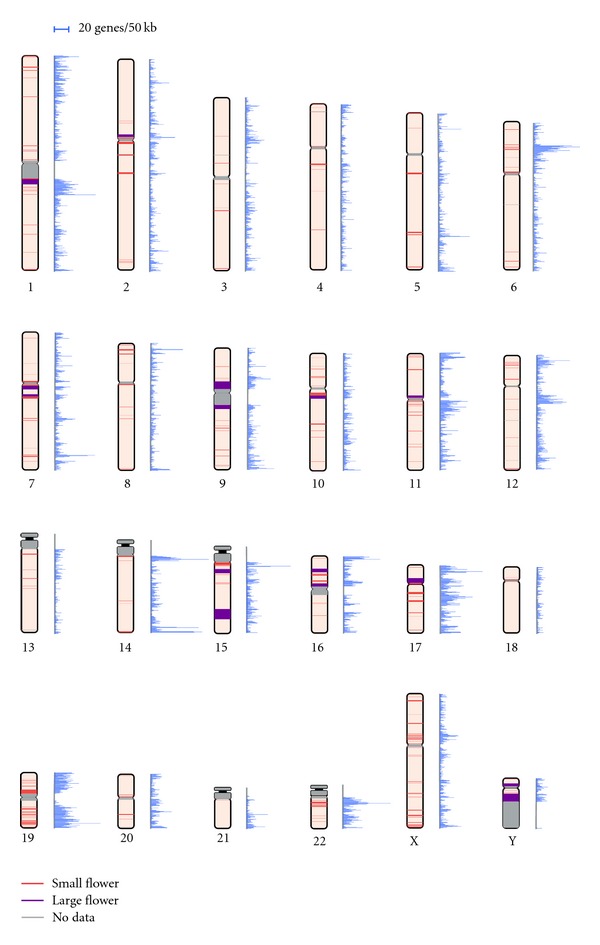
Distribution of Flowers and gene density on chromosomes. A bar on a chromosome denotes the position of a Flower and the width of the bar represents the Flower length. The color of the bar represents the patterns of Flowers: small (red) and large (purple) Flowers. Gray bars stand for no genomic sequence data. Mapping of the bars was accomplished using *ColoredChromosomes* [11]. Gene density is represented alongside each chromosome. The scale for the gene density is placed at the top of the figure.

We scanned the entire human genome and, based on our definitions, detected 291 Flowers (Figure 2). The Flowers were composed of at least two duplications with some degree of similarity and were more than 50 kb in length (see Section 2). Summary statistics for the 291 Flowers are shown in Table 1 (detailed information is presented in Table S1 in Supplementary Material available online at doi:10.1155/2012/917678).

The sum of the 291 Flower lengths was approximately 179 Mb, which comprised 6% of the genome. The mean length of a Flower was 615 kb and the median length was 210 kb (Table 1). The mean number of copy units in a Flower was approximately three and the mean length of a copy unit was 50 kb. Most Flowers (87%) were shorter than 1 Mb, and only 5% of Flowers exceeded 3 Mb (Figure S1). We categorized Flowers into two groups to distinguish exceptionally large Flowers and then fixed the 5% cutoff point (at 3 Mb) in the Flower length distribution (large Flowers (≥3 Mb, 15 Flowers) and small Flowers (<3 Mb, 276 Flowers). Compared with small Flowers, large Flowers were composed of more complicated structures. The mean number of copy units was 7.3, more than twice that of small Flowers (3.1). Interestingly, 10 of the 15 large Flowers were located in pericentromeric regions or near heterochromatin regions (Figure 2). This finding is consistent with the previous observation of numerous segmental duplications in pericentromeric regions [8].

The largest Flower (11.7 Mb), however, was located on a euchromatic region on chromosome 15. The structure of the Flower was extremely complicated, with many duplication units, as represented in Figure S2. A part of this region is likely responsible for a recurrent microdeletion syndrome [13]. Four unrelated patients with this syndrome shared breakpoints for *de novo* 1.7 to 3.9 Mb deletions in the region. The deletions could be the result of recurrent nonallelic homologous recombination between highly similar duplicates. Additionally, a polymorphic 1.2-Mb inversion has also been mapped to the same region [14]. Even though such dynamic rearrangements occur, this region contains 46 protein-coding genes.

 Two classes of Flowers might be different types of genomic structures in mechanisms for generating or maintaining Flowers. Large Flowers were usually located in pericentromeric regions, and small Flowers were located in euchromatic regions. Since Large Flowers showed a more complicated structure than small Flowers, they have likely experienced more dynamic rearrangements compared to small Flowers.

### 3.2. Flower Distribution and Gene Density

Flowers were distributed nonuniformly over the human genome (Figure 2 and Table 2). The pattern of distribution was different not only in the location on a chromosome, but also between the 24 chromosomes. The arithmetic mean of Flower density (i.e., the number of Flowers per 1 Mb) for a chromosome was 0.12 ± 0.10 (Table 2). Most chromosomes showed a relatively similar density to one another (Table 2), with the exception of chromosome 19. The gene density of chromosome 19 (0.47) was the largest, and about 20% of the length of the chromosome was occupied by 26 Flowers.

Previous studies suggested that genomic structures, such as segmental duplications or CNVs, are often observed in gene-rich regions [15–18]. A possible reason for the high Flower density in chromosome 19 might be related to the fact that the density of genes in the chromosome is high. In fact, the association between Flowers and their gene content was also observed. The gene density of each chromosome seemed to be associated with the location of Flowers (Figure 2). To evaluate this association, we compared the gene density in 277 Flowers (*D* length < 1 Mb) with the gene density in randomly selected regions from the genome (Table 3). The mean gene density in a Flower was 0.61 per 10 kb, more than five times the value for the randomly selected regions (i.e., 0.12; *P* < 10^−5^, *Z*-test). Also, the density of protein-coding genes, 0.34, was 3.4 times larger than that of the randomly selected regions (0.09; *P* < 10^−5^, *Z*-test). The gene density of 14 large Flowers (*D* length > 1 Mb) was 0.35, larger than that of the randomly selected regions (data not shown). Therefore, we concluded that Flowers are generich relative to other regions of the genome.

### 3.3. Flower Genes

Among all Flowers, 82% included at least one gene. We found 2,844 genes in these Flowers, and these genes comprised 8% of the human genes annotated in NCBI build 36. Among these, there were 1,417 protein-coding genes (50%), 1,085 pseudogenes (38%), and 116 RNA genes (4%) (Table S2). The remaining 226 (8%) were “unknown” or “other” categories in the NCBI annotation. To examine the functions of the genes in Flowers, we classified the genes according to the GO categories. To detect biases in the functions of these genes compared to the entire set of human genes, we compared the observed gene number of Flowers in each given category with the expected number. The expected gene numbers were calculated based on the proportion of human genes in each category (Table S3).  Table 4 shows GO categories that were significantly overrepresented in Flowers (*P* < 10^−4^, hypergeometric test).

The most overrepresented category was the alpha-amylase multigene family (*AMY*), which is located on chromosome 1. The family consists of *AMY1* and *AMY2*. Amylases catalyze the breakdown of starch and glycogen into disaccharides or trisaccharides, and the genes are highly expressed in the salivary gland and pancreas. *AMY1* showed extensive CNVs that are known to be present among several ethnic populations. The copy number, however, did not depend on the geographic distribution of populations, but instead was associated with differences in diets. Populations with high-starch diets have a larger number of *AMY1* gene copies than those with low-starch diets [19, 20]. The number of *AMY1 *gene copies correlates with the amount of AMY1 proteins in saliva [20].

The second most frequent category in Table 4 was glucuronosyltransferase activity, which includes the UDP glucuronosyltransferase (*UGT*) multigene family. Members of the *UGT* gene family are divided into two subfamilies, *UGT1* and *UGT2.* They are located on 2q37 and 4q13-13.2 and in one and two Flowers, respectively. *UGT*s encode enzymes that catabolize small lipophilic molecules, such as steroids, bilirubins, hormones, drugs, environmental toxicants, and carcinogens, into water-soluble glucuronides [21]. A mature *UGT1* mRNA is composed of five exons. However, depending on the substrate, several distinct mRNAs are observed. Interestingly, this variety in mRNAs is caused by alternative splicing. The region for the *UGT1* subfamily on chromosome 2 encodes 13 sets of exon 1 and a single set of exon 2 to exon 5. Splicing of each variable exon (exon 1) to the four constant exons (exon 2 to 5) generates diverse functional *UGT1 m*RNA. Nine of the 13 first exons encode the specific N-terminal domains, conferring the substrate specificity of the enzyme [22], whereas the remaining four are pseudogenized. Similar to *UGT1*, the region for the protocadherin beta (*PCDHB*) gene family encodes 16 different proteins with variable N-termini. In contrast to *UGT1*, each PCDHB protein is encoded by a single exon, and in total, 16 independent exons exist in this region. *PCDHB* is also in an overrepresented GO category in Flowers (see Table 4).

 Except for *AMY* and *PCDHB*, most overrepresented GO categories in Table 4 are related to immune responses (multigene families of immunoglobulin, major histocompatibility complex (*MHC*), and defensin) and detoxifications (multigene families of *UGT*, glutathione S-transferase, and cytochrome P450). This is consistent with previous results for segmental duplications and CNVs showing that these regions are rich in genes that can interact with their environment [23, 24].

### 3.4. Gene Conversion within a Flower

We calculated the minimum and maximum identities between copies of a gene in a Flower to investigate the age of Flowers. We can estimate the time of occurring of the oldest and most recent duplications, from the minimum and maximum identities, respectively. For example, the lowest minimum identity was 77.7% (Table 1), suggesting that the duplication likely occurred prior to the placental mammal radiation. In the distributions of minimum and maximum identities, the minimum identity was evenly distributed from low to high identity (78% ~ 100%), whereas the maximum identity was concentrated at 100% identity (Figure S3). One hundred Flowers (34%) had exactly identical copies and 55 Flowers (19%) had almost identical copies (i.e., 99% to <100%). When we considered all the BLAST hits in Flowers, the number of hits with ≥99% identity was larger than that with <99% identity (Figure S4).

**Figure 3:**
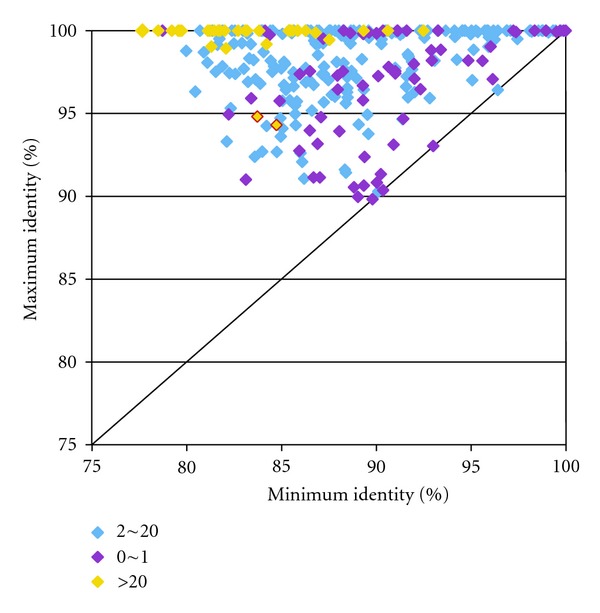
Scatter plot of maximum and minimum identity. The *X* and *Y* axes show the maximum and minimum percent identity of blast hits in a Flower. A dot represents one Flower. The 291 Flowers were classified into three groups, based on the number of Flower genes: 0 ~ 1, 2 ~ 20, and more than 20, are colored purple, blue, and yellow, respectively. The two yellow dots outlined in red are two exceptional Flowers with low maximum identity.

Interestingly, the maximum identity did not depend upon the values of the minimum identities. Many old Flowers, which had rather low minimum identities, showed 100% maximum identity (Figure 3). In addition, we found that the maximum identity depended upon the number of genes included in a Flower. Flowers containing up to 20 genes showed variety in maximum identities, whereas Flowers containing more than 20 genes showed almost 100% maximum identity (Figure 3). This suggests that the number of gene copies is associated with the genetic distance between paralogs within a Flower.

This observation suggested one hypothesis to explain the association of gene copy numbers with the large number of highly similar copies in Flowers: gene conversions occurred between gene copies, generating identical sequences within a Flower. The conversion is likely to be enriched in the case of a large number of gene copies. To examine the possibility of gene conversions within a Flower, we performed a gene conversion detection test, using the CHAP package [12]. This method identifies the orthologous and paralogous sequences within input sequences and detects regions showing significantly higher identity between the paralogs rather than between the orthologs. This pipeline was developed to analyze gene cluster regions and was useful for the detection of gene conversions within Flowers. We applied this method and obtained results for 189 Flowers. There were some technical difficulties in producing results for all Flowers (see Section 2).

Among the 189 Flowers, gene conversion events that occurred in the human lineage were detected in 157 Flowers (83% of the tested Flowers). In the entire duplicated regions in the 157 Flowers, the average number of gene conversion events was 21, which corresponds to 20% of the duplicated regions (Table 5). Furthermore, in 798 genes of the 189 tested Flowers, 67% (533 genes) had experienced conversion. On average, 49% of the converted regions in a Flower had overlapped gene regions. In the 533 genes with conversions, the average number of conversions per gene was 6, and the proportion of converted region in the gene region was 21% (Table 5). These results indicate that gene conversions have been occurring frequently and recently within a Flower, especially in the gene regions, but also in the intergenic regions. Gene conversion events could play an important role in the evolution of Flower genes.

### 3.5. Simulations of Gene Conversions

**Figure 4:**
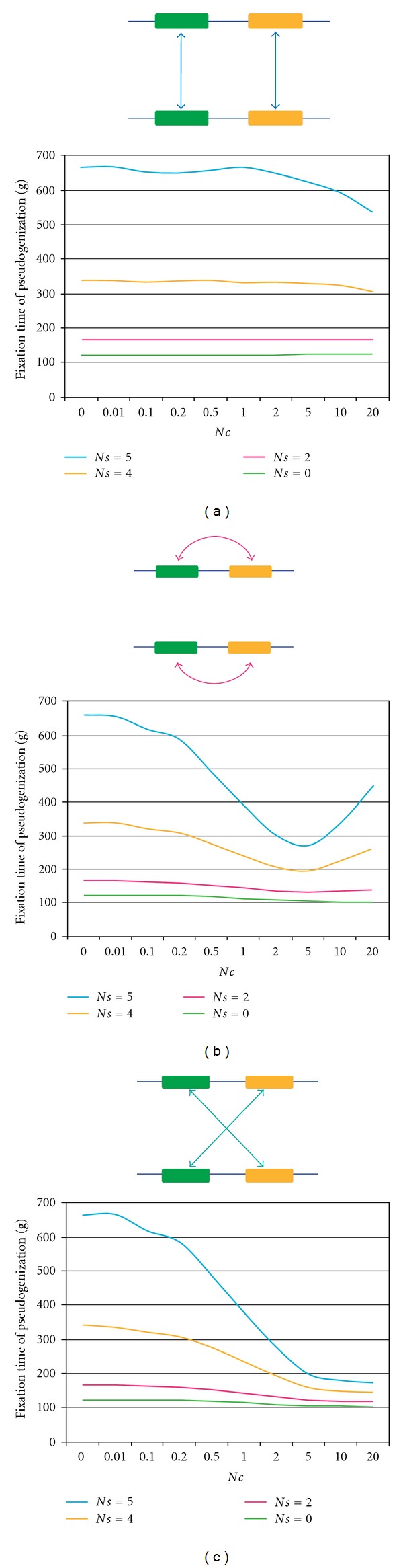
Fixation time of pseudogenes. We tested three types of gene conversion (double-headed arrows): (a) allelic-*trans*, (b) *cis*, and (c) nonallelic-*trans* gene conversion. The three graphs show that the pseudogene fixation time (in units of a generation) in a population (2*N* = 100) depends on the gene conversion rate (*Nc* = 0–20). The mutation rate is assumed to be constant (2*Nμ* = 1) for the three cases shown here.

To understand the effects of gene conversion on the evolution of Flower genes, we performed simulation studies (for details, see Section 2). The null hypothesis was that the fixation time of a pseudogene at a locus does not depend upon the rates of gene conversion. We measured the fixation time of a pseudogene in a population under neutrality or negative selection against fixation of pseudogenes in a diploid individual population. We assumed that gene conversion between alleles at two loci can take place in *any* of three different schemes: allelic-trans, allelic-cis, and nonallelic-trans (Figure 4). In all cases, under neutrality, the fixation time of a pseudogene did not appear to depend upon the rate of gene conversion (*Ns* = 0, Figure 4). Introduction of the purifying selection (*Ns* ≥ 2) generally increased the fixation time with a constant *Nc*, and if *Nc* increases the fixation time gradually decreased. Nevertheless, in the case of cis gene conversion, when the conversion rate was lower than the mutation rate (*Nμ* = 0.5 and *Nc* ≤ 5), the fixation time decreased when *Nc* increased, but when *Nc* ≥ 5, the fixation time increased. The life span of a multigene family in a Flower seems to be extended by gene conversion (in cis), probably because frequent gene conversion enhances homogenization of functional paralogs and thus counteracts pseudogenization. Frequent conversion also helps to convert pseudogenes to functional genes.

## 4. Discussion

In the human genome, we detected 291 regions with particular genomic structures that we termed “Flowers.” Based on our characterization of the Flowers, we can draw two main conclusions. First, genes appear to be enriched in Flowers. Second, there is evidence of frequent gene conversion between duplicates within Flowers. These two findings may sometimes be contradictory, because gene conversion from a pseudogene has the potential to be deleterious for functional genes. Genomic rearrangements associated with several inherited diseases could be caused by this kind of gene conversion from paralogous pseudogenes [25]. In our observations, 30% of Flower genes were pseudogenes (Table 2 and Table S1).

The results of this study, on the other hand, showed that frequent gene conversion could play an important role in the preservation of functionality of multigene families. In Figure 3, the 32 Flowers that had over 20 genes showed higher maximum identity than Flowers with fewer genes. Flowers containing a large copy number of genes have likely experienced more frequent gene conversions than other Flowers, and the gene copies had been homogenized. However, there were two exceptional Flowers (yellow dots outlined in red in Figure 3), which contained a large number of genes, but showed a maximum identity of less than 95%. These exceptions included immunoglobulin lambda variable (*IGLV*) and major histocompatibility complex class I (*MHC*) genes. These families need to maintain genetic diversity, which is important for their functions. This result implies that the *MHC* and immunoglobulin gene families have evolved under purifying selection against homogenization between members of a multigene family [26].

A similar example of purifying selection was observed in melanoma antigen family A (*MAGE-A*) in a Flower on the X chromosome [27]. Members of this multigene family are expressed in cancer cells, encode epitopes recognized by *MHC*, and are associated with cancer immunity [28]. The genetic diversity between the *MAGE-A3 *and* A6* genes has likely been preserved by purifying selection against homogenization to maintain association with a particular MHC molecule.

In this study, we characterized several biologically significant features of genomic structures, called Flowers, in the human genome. First, large-sized and complex Flowers were usually located in pericentromeric regions. In primates, the genomic structures of pericentromeric regions resulted from numerous segmental duplications of euchromatin regions [8, 29]. Recent reports suggest that complex rearrangements played a role in the deterioration of functional genes during the generation of novel centromeres [30, 31].

Second, we suggested an evolutionary mechanism for the preservation of multigene families within Flowers. Simulation studies showed that frequent gene conversion, probably at a higher rate than the mutation rate, could extend the age (lifespan) of a gene family. Negative selection against pseudogenization was a likely driving force in the maintenance of a multigene family.

Third, we provided evidence for the interrelationship of Flowers with the functions of multigene families. Multigene families related to immune responses and detoxification were overrepresented in the Flowers. These gene families have evolved under purifying selection opposing gene conversion to maintain the genetic variety between paralogous genes. Frequent genomic rearrangements in Flowers could drive the duplication of genes and increase the number of genes. This evolutionary mode was likely to be more favorable for multigene families related to immune responses and detoxification, which need to adapt to environmental changes.

In conclusion, a Flower has an important role in the evolution of multigene families. Future studies will be extended to the genomes of other organisms to further understand the evolution of multigene families contained in genomic structures. 

## Supplementary Material

Table S1. List of the 291 Flowers identified in the present study.
Table S2. List of genes contained in Flowers.
Table S3. List of GO id of genes contained in Flowers.
Figure S1. Distribution of Flower length.
Figure S2. Dot-plot of the largest Flower.
Figure S3. Distribution of maximum and minimum identity.
Figure S4. The number of BLAST hits, categorized by percent identity.

## Figures and Tables

**Table 1:** Statistical data for 291 Flowers. Definitions of each term are described in Section 2.

*N* = 291	Sum	Mean	S.D.	Median	Min.	Max.
Flower length (Kb)	179,197	615	1,349	210	51	11,695
*D* length/Flower length		0.33	0.22	0.27	0.01	0.96
Effective number of copy units		3	3	3	2	22
Copy unit length (Kb)		50	95	23	1	1,122
Length of inverted copy/*D* length		0.4	0.4	0.5	0.0	1.0
Minimum identity (%)		88.5	5.0	88.0	77.7	100.0
Maximum identity (%)		98.0	2.6	99.2	89.8	100.0
Gene region length/*D* length		0.4	0.3	0.4	0.0	1.0
Gene number^a^	2,844	10	19	4	0	133
Pseudogene number/gene number^a^		0.3	0.3	0.3	0.0	1.0

^a^The number of Flower genes.

**Table 2:** Statistics of Flowers in each chromosome.

Chr.	Flower number	Flower number /1Mb	Flower length^a^ (Kb)	Copy unit length^a^ (Kb)	Effective number of copy units^a^	Min. identity^a^ (%)	Max. identity^a^ (%)	Gene number^a,b^	Pseudogene number/Gene number ^a,b^
1	27	0.12	472	80	3.3	89.8	98.7	12	0.3
2	10	0.04	1,110	102	2.8	86.5	98.8	19	0.3
3	6	0.03	271	17	2.3	88.0	96.4	4	0.7
4	10	0.05	346	47	3.5	88.2	97.6	6	0.4
5	9	0.05	556	39	5.7	88.0	96.5	9	0.2
6	15	0.09	238	39	2.5	88.1	97.2	6	0.3
7	17	0.11	789	54	3.0	88.8	97.6	12	0.4
8	11	0.08	373	40	3.4	87.6	97.2	14	0.3
9	12	0.10	1,308	129	3.2	88.3	97.2	17	0.3
10	14	0.11	707	74	2.7	90.3	97.5	8	0.2
11	18	0.14	405	26	4.0	87.5	96.8	8	0.5
12	12	0.09	223	18	2.4	88.7	96.4	2	0.1
13	6	0.06	264	24	2.1	90.2	97.3	2	0.4
14	6	0.07	449	75	2.6	86.8	99.1	20	0.5
15	7	0.09	2,911	141	3.8	87.1	99.9	27	0.4
16	10	0.13	1,082	85	3.0	89.1	99.3	13	0.4
17	11	0.14	1,031	73	2.7	91.4	99.2	12	0.3
18	3	0.04	268	16	4.1	87.5	99.0	1	0.0
19	26	0.47*	422	23	3.9	86.5	97.0	7	0.2
20	5	0.08	374	35	2.7	86.1	98.5	2	0.0
21	1	0.03	94	5	2.0	95.6	98.2	1	1.0
22	10	0.29*	351	31	2.4	90.3	98.3	11	0.3
X	39	0.26	270	28	3.8	89.1	99.2	5	0.2
Y	6	0.23	2,319	110	6.1	88.6	99.9	30	0.6

Mean	12	0.12	693	52	3.5	88.7	98.0	10	0.3
S.D.	9	0.10	680	37	1.2	2.0	1.1	8	0.2
Mean w/o XY	11	0.11	638	51	3.3	88.7	97.9	10	0.3
S.D. w/o XY	7	0.10	607	37	0.9	2.1	1.0	7	0.2

^a^Mean values of each chromosome, ^b^Flower genes.

*P < 0.05 by *Z*-test.

**Table 3:** Comparison of gene density of Flowers with randomly selected regions. Except Flowers with *D* length ≥ 1 Mb, the number of Flower genes was compared to that of 1,000 randomly selected regions on the human genome.

		Gene number in 277 Flowers	Gene number/10 Kb	Protein-coding gene number	Protein-coding gene number/10 kb
Flower		1,841	0.61*	1,030	0.34*

Randomly selected region (*n* = 1,000)	Mean	371	0.12	282	0.09
	S.D.	39	0.01	30	0.01
	Min.	272	0.09	204	0.07
	Max.	561	0.19	439	0.15

*P = 0, *Z*-test.

**Table 4:** Functions of genes in Flowers. For a GO category, we tested significance of frequency of Flower genes compared to total number of the human genes. This table represents GO categories showing *P* < 10^−4^, the observed number of Flower genes ≥3, and the ratio of observation to expectation ≥3.

GO: ID	Detail	Catalog	Observation^a^	Obs/Exp^b^	Multigene families on Flowers
GO: 0004556	Alpha-amylase activity	Function	3	21.1	Amylase alpha

GO: 0015020	Glucuronosyltransferase activity	Function	15	15.0	UDP glucuronosyltransferase

GO: 0019864	IgG binding	Function	7	14.7	Fc fragment of IgG

GO: 0016339	Calcium-dependent cell-cell adhesion	Process	12	10.5	Protocadherin beta

GO: 0003823	Antigen binding	Function	19	10.0	Immunoglobulin, leukocyte immunoglobulin-like receptor, killer cell immunoglobulin-like receptor

GO: 0004364	Glutathione transferase activity	Function	9	9.0	Glutathione S-transferase

GO: 0019882	Antigen processing and presentation	Process	13	8.6	Major histocompatibility complex, class I, MHC class I polypeptide-related sequence, retinoic acid early transcript, UL16-binding protein, C-type lectin domain family

GO: 0006805	Xenobiotic metabolic process	Process	10	8.4	UDP glucuronosyltransferase, defensin, alpha, aldo-keto reductase family

GO: 0006952	Defense response	Process	17	4.9	Interferon, alpha, leukocyte immunoglobulin-like receptor, pregnancy-specific beta-1-glycoprotein, major histocompatibility complex, class I, SP140 nuclear body protein

GO: 0032312	Regulation of ARF GTPase activity	Process	7	4.8	ArfGAP with GTPase domain, centaurin, gamma-like family

GO: 0020037	Heme binding	Function	24	4.4	Cytochrome P450, nitric oxide synthase, HECT domain and RLD

GO: 0007565	Female pregnancy	Process	10	4.3	Pregnancy-specific beta-1-glycoprotein

GO: 0005792	Microsome	Component	33	3.9	UDP glucuronosyltransferase, cytochrome P450, flavin-containing monooxygenase, hydroxy-delta-5-steroid dehydrogenase

GO: 0042742	Defense response to bacterium	Process	12	3.2	Defensin, alpha, defensin, beta, MHC class I polypeptide-related sequence

GO: 0009615	Response to virus	Process	12	3.1	Defensin, alpha, interferon, alpha, chemokine (C-C motif) ligand, leukocyte immunoglobulin-like receptor

^a^The observed number of Flower genes for a GO category.

^b^The ratio of the observed number of Flower genes to the expected number of genes.

**Table 5:** Statistics of detected gene conversion events within a Flower. The average and standard deviation of several values in the 157 Flowers and 533 genes, experiencing gene conversion events in the human lineage.

157 Flowers	No. of events /Flower	Prop. of converted /*D* region	Prop. of gene regions in the converted region	No. of genes /Flower	No. of converted genes/Flower
Average	21	0.25	0.49	5	3
S.D.	29	0.31	0.35	5	3

533 Genes	No. of events/gene		Prop. of converted/gene		

Average	6		0.21		
S.D.	20		0.30		

## References

[1] Huminiecki L, Wolfe KH (2004). Divergence of spatial gene expression profiles following species-specific gene duplications in human and mouse. *Genome Research A*.

[2] Blekhman R, Oshlack A, Gilad Y (2009). Segmental duplications contribute to gene expression differences between humans and chimpanzees. *Genetics*.

[3] Mefford HC, Eichler EE (2009). Duplication hotspots, rare genomic disorders, and common disease. *Current Opinion in Genetics and Development*.

[4] Ayala FJ, Coluzzi M (2005). Chromosome speciation: humans, Drosophila, and mosquitoes. *Proceedings of the National Academy of Sciences of the United States of America*.

[5] Skaletsky H, Kuroda-Kawaguchi T, Minx PJ (2003). The male-specific region of the human Y chromosome is a mosaic of discrete sequence classes. *Nature*.

[6] Bhowmick BK, Satta Y, Takahata N (2007). The origin and evolution of human ampliconic gene families and ampliconic structure. *Genome Research*.

[7] Rozen S, Skaletsky H, Marszalek JD (2003). Abundant gene conversion between arms of palindromes in human and ape Y chromosomes. *Nature*.

[8] She X, Horvath JE, Jiang Z (2004). The structure and evolution of centromeric transition regions within the human genome. *Nature*.

[9] Linardopoulou EV, Williams EM, Fan Y, Friedman C, Young JM, Trask BJ (2005). Human subtelomeres are hot spots of interchromosomal recombination and segmental duplication. *Nature*.

[10] Ohtsubo Y, Ikeda-Ohtsubo W, Nagata Y, Tsuda M (2008). GenomeMatcher: a graphical user interface for DNA sequence comparison. *BMC Bioinformatics*.

[11] Böhringer S, Gödde R, Böhringer D, Schulte T, Epplen JT (2002). A software package for drawing ideograms automatically. *Online Journal of Bioinformatics*.

[12] Song G, Hsu C-H, Riemer C (2011). Conversion events in gene clusters. *BMC Evolutionary Biology*.

[13] Sharp AJ, Selzer RR, Veltman JA (2007). Characterization of a recurrent 15q24 microdeletion syndrome. *Human Molecular Genetics*.

[14] Antonacci F, Kidd JM, Marques-Bonet T (2009). Characterization of six human disease-associated inversion polymorphisms. *Human Molecular Genetics*.

[15] Zhang L, Lu HHS, Chung WY, Yang J, Li WH (2005). Patterns of segmental duplication in the human genome. *Molecular Biology and Evolution*.

[16] She X, Liu G, Ventura M (2006). A preliminary comparative analysis of primate segmental duplications shows elevated substitution rates and a great-ape expansion of intrachromosomal duplications. *Genome Research*.

[17] Nguyen DQ, Webber C, Hehir-Kwa J, Pfundt R, Veltman J, Ponting CP (2008). Reduced purifying selection prevails over positive selection in human copy number variant evolution. *Genome Research*.

[18] Nguyen DQ, Webber C, Ponting CP (2006). Bias of selection on human copy-number variants. *Plos Genetics*.

[19] Groot PC, Mager WH, Frants RR (1991). Interpretation of polymorphic DNA patterns in the human *α*-amylase multigene family. *Genomics*.

[20] Perry GH, Dominy NJ, Claw KG (2007). Diet and the evolution of human amylase gene copy number variation. *Nature Genetics*.

[21] Tukey RH, Strassburg CP (2000). Human UDP-glucuronosyltransferases: metabolism, expression, and disease. *Annual Review of Pharmacology and Toxicology*.

[22] Gong Q-H, Cho JW, Huang T (2001). Thirteen UDPglucuronosyltransferase genes are encoded at the human UGT1 gene complex locus. *Pharmacogenetics*.

[23] Bailey JA, Gu Z, Clark RA (2002). Recent segmental duplications in the human genome. *Science*.

[24] Cooper GM, Nickerson DA, Eichler EE (2007). Mutational and selective effects on copy-number variants in the human genome. *Nature Genetics*.

[25] Chen JM, Cooper DN, Chuzhanova N, Férec C, Patrinos GP (2007). Gene conversion: mechanisms, evolution and human disease. *Nature Reviews Genetics*.

[26] Nei M, Rooney AP (2005). Concerted and birth-and-death evolution of multigene families. *Annual Review of Genetics*.

[27] Katsura Y, Satta Y (2011). Evolutionary history of the cancer immunity antigen MAGE gene family. *Plos ONE*.

[28] Van der Bruggen P, Traversari C, Chomez P (1991). A gene encoding an antigen recognized by cytolytic T lymphocytes on a human melanoma. *Science*.

[29] Horvath JE, Gulden CL, Vallente RU (2005). Punctuated duplication seeding events during the evolution of human chromosome 2p11. *Genome Research*.

[30] Ventura M, Antonacci F, Cardone MF (2007). Evolutionary formation of new centromeres in macaque. *Science*.

[31] Lomiento M, Jiang Z, D’Addabbo P, Eichler EE, Rocchi M (2008). Evolutionary-new centromeres preferentially emerge within gene deserts. *Genome Biology*.

