# Supplementary material for: Genomic Structure and Evolution of Multigene Families: “Flowers” on the Human Genome
Source: Int J Evol Biol. 2012 Jun 19;2012:917678. doi: 10.1155/2012/917678 (PMC3388347; doi:10.1155/2012/917678)

Supplemental Material

Table S1. List of the 291 Flowers identified in the present study.

Table S2. List of genes contained in Flowers.

Table S3. List of GO id of genes contained in Flowers.

*The above three supplemental tables were separated Excel files.

Figure S1. Distribution of Flower length

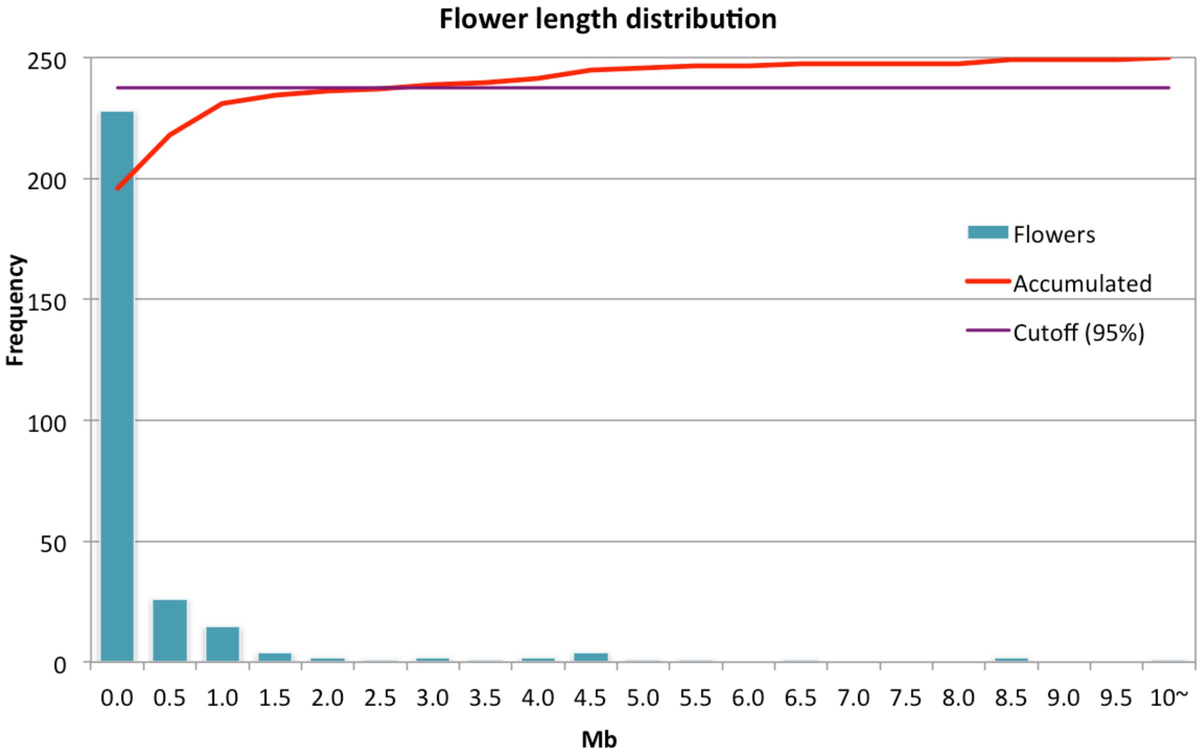

Figure S2. Dot-plot of the largest Flower

The Flower is located on chromosome 15, 72.1-83.8 Mb. A line in the figure indicates a BLAST analysis 'hit' and the colors of the lines show the percent identities (see key on the right side). At the top and right of the dot-plot, a pink arrow indicates loci and length of genes. The blue arrow indicates loci in a region that shows up as a recurrent microdeletion.

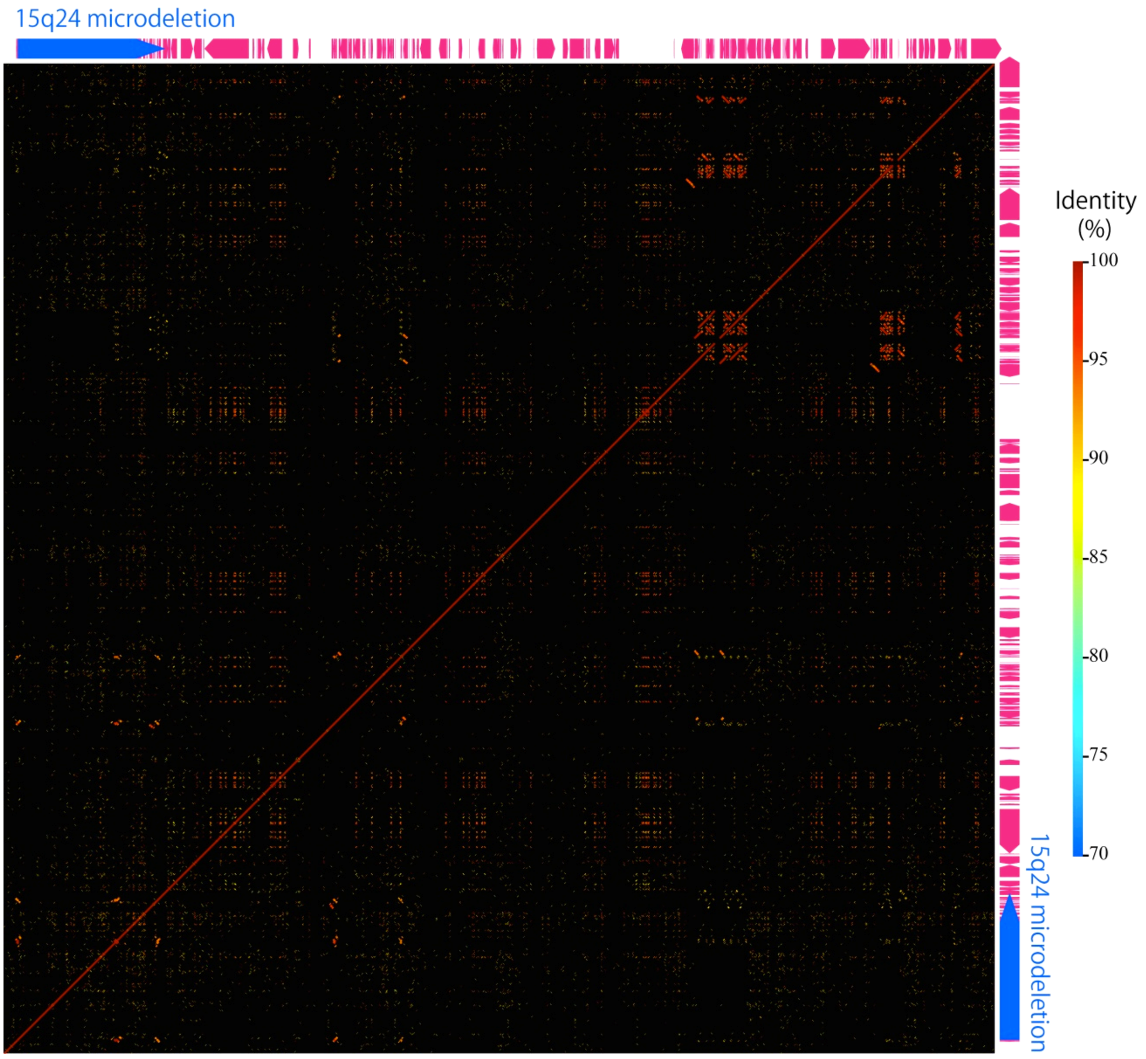

Figure S3. Distribution of maximum and minimum identity

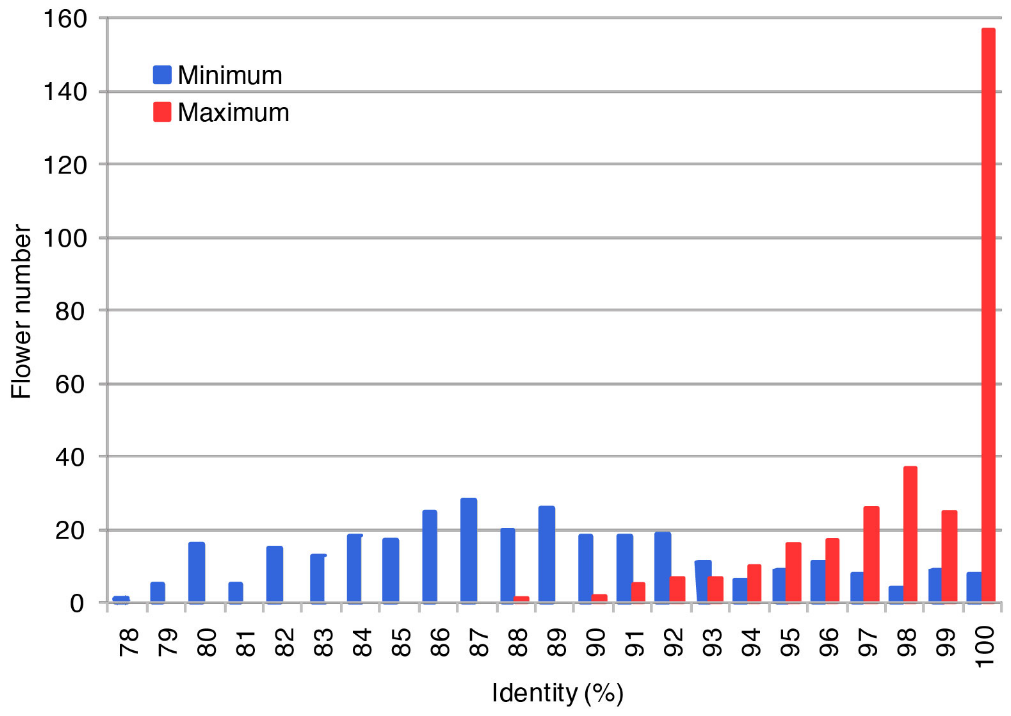

Figure S4. The number of BLAST hits, categorized by percent identity.

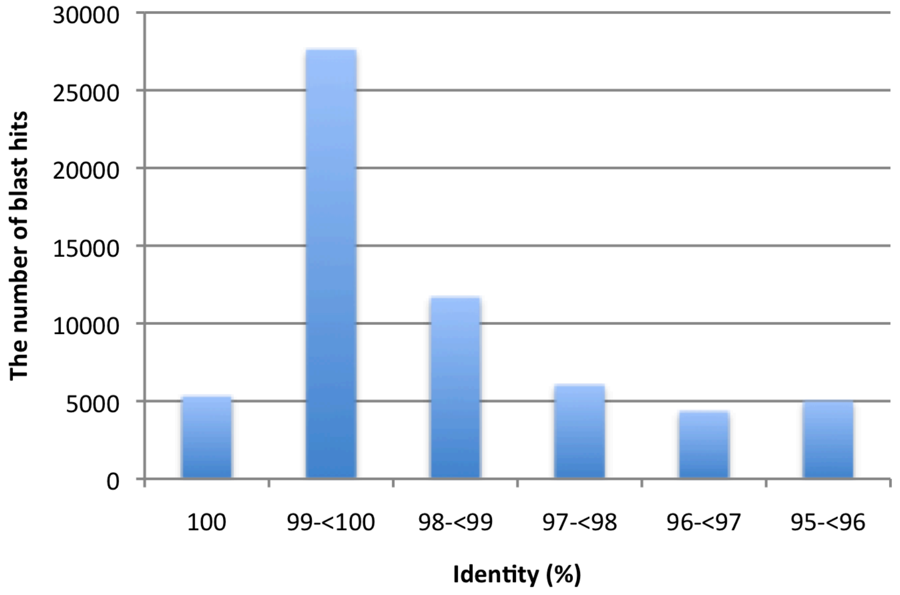

Supplement: Supplementary file 1 — Table S1. List of the 291 Flowers identified in the present study. Table S2. List of genes contained in Flowers. Table S3. List of GO id of genes contained in Flowers. Figure S1. Distribution of Flower length. Figure S2. Dot-plot of the largest Flower. Figure S3. Distribution of maximum and minimum identity. Figure S4. The number of BLAST hits, categorized by percent identity. [file 917678.f1.pdf]
